# A Rare Presentation of Concurrent *Scedosporium apiospermum* and *Madurella grisea* Eumycetoma in an Immunocompetent Host

**DOI:** 10.1155/2012/154201

**Published:** 2012-10-22

**Authors:** Vivek Gulati, Seun Bakare, Saket Tibrewal, Nizar Ismail, Junaid Sayani, Davinder Paul Singh Baghla

**Affiliations:** ^1^Department of Orthopaedic, Ealing Hospital NHS Trust, Uxbridge Road, Southall UB1 3HW, UK; ^2^HPA Mycology Reference Laboratory, Myrtle Road, Bristol BS2 8EL, UK

## Abstract

Mycetoma is a disfiguring, chronic granulomatous infection which affects the skin and the underlying subcutaneous tissue. We present an atypical case of recurrent mycetoma without ulceration, in a 35-year-old immunocompetent male caused by *Scedosporium apiospermum* sensu stricto and *Madurella grisea*, occurring at two separate anatomical sites.

## 1. Introduction

Mycetoma is a localized, chronic, progressive granulomatous condition affecting skin, subcutaneous tissue, and bone. Individuals classically present with a triad of tumefaction, suppuration, and ulceration. The highest incidence occurs between the ages of 20 to 40 [1, 2] with a male-to-female ratio of 5 : 1 [3]. The implicated organisms are saprophytic fungi (eumycetoma) or non-acid-fast anaerobic filamentous bacteria (actinomycosis), at a ratio of 2 : 3 [1]. 

Infection follows traumatic implantation of microorganisms into the skin of the hands or feet (70–80%) most commonly. A granulomatous inflammatory response in the deep dermis and the subcutaneous tissue develops. The disease is endemic to tropical and subtropical regions, known as the mycetoma belt (Sudan, India, Yemen, Colombia, and others) [2]. Occupational exposure includes agricultural workers handling contaminated vegetation. Chronic neglected infection may be disfiguring, leading to deformity and amputation. Differential diagnoses include cutaneous tuberculosis, coccidioidomycosis, and bone and soft tissue tumors.

To our knowledge this is the first report of a multifocal mycetoma caused by 2 separate fungal species in distinct anatomical locations in an immunocompetent host.

## 2. Case Presentation

A 35-year-old gentleman of Indian origin, who works as a factory vegetable sorter in London, presented in February 2008 with a two-month history of a painless lump over the dorsum of his right hand. There was no history of trauma, and the patient reported no systemic symptoms. Although the gentleman had emigrated from India 11 years ago, he denied any recent travel. Past medical history and family history were both unremarkable. He subsequently underwent a surgical excision biopsy but was lost to followup. 

The gentleman then represented eight months later with recurrence of the previously excised hand swelling but in addition noticed a new painless swelling on the dorsum of his right ankle. Both lesions were functionally asymptomatic and no other systemic symptoms were reported. He was previously well with no past history of other infections.

**Figure 1:**
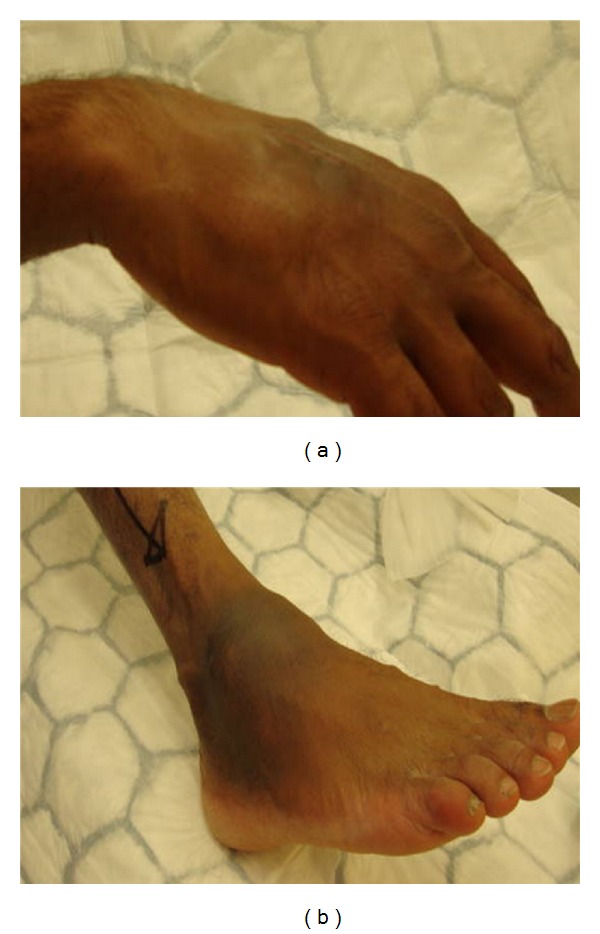
Nodular appearance of right dorsal hand swelling (a) and foot (b).

Clinical examination of the right anterolateral ankle demonstrated an 8 × 5 cm firm, nonfluctuant, nontender lump, which was tethered to deep structures and adherent to the hyperpigmented overlying skin (Figure 1). The lesion was normothermic and displayed no regional lymphadenopathy. 

His right hand revealed a well-healed midline surgical scar on the dorsal aspect, adjacent to a dorsal-ulnar 6 × 5 cm nodular, soft, fluctuant, yet non-tender lump. Tethering to overlying skin and deep tissues was noted; there was no regional lymphadenopathy (Figure 1). 

Investigations revealed a normal differential white cell count, a normal immunoglobulin levels, and a normal CD4, CD8 cell counts; he tested negative for HIV. No abnormalities were present on biochemical laboratory studies including CRP.

**Figure 2:**
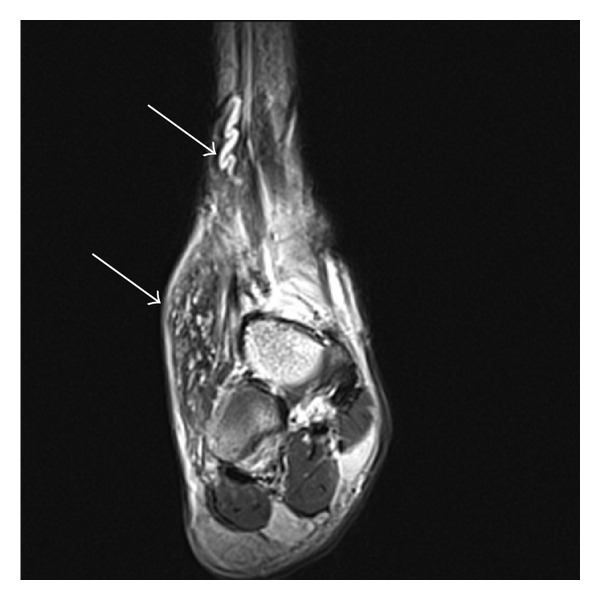
Coronal T1 MRI of right foot large subcutaneous soft tissue lesion anterolaterally (arrow) with a large prominent proximal feeding vessel (arrow).

Plain radiographs confirmed no underlying bony abnormality or periosteal reaction. T1 weighted magnetic resonance imaging of the ankle lesion showed a 6 × 2 cm heterogenous soft tissue lesion, with scattered cystic regions, prominent vasculature, and surrounding soft tissue oedema, suspicious of either a vascular malformation or a neoplasm (Figure 2). 

**Figure 3:**
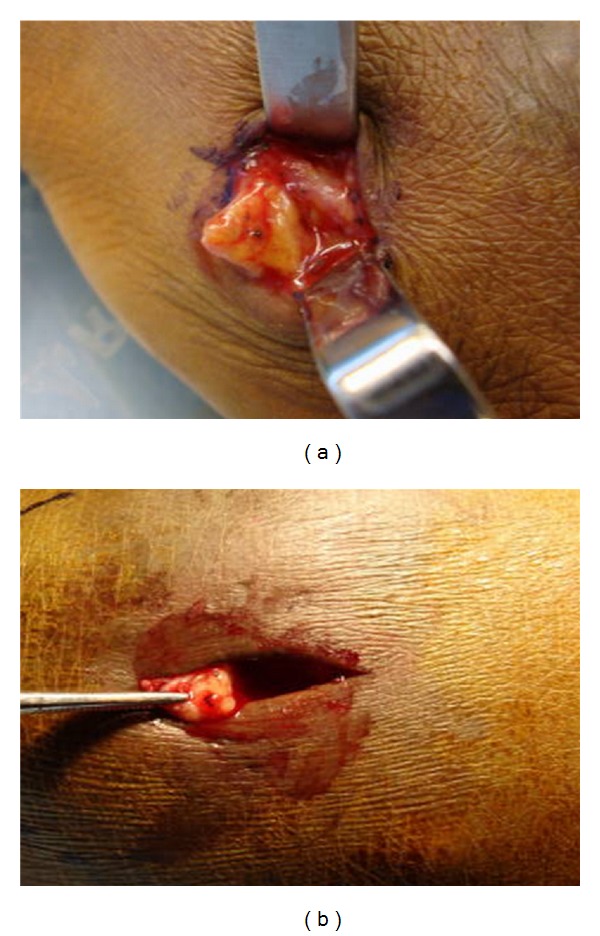
Intraoperative photographs of the right hand (a) and the right foot (b) demonstrating multiple black granules.

Retrospective histopathological assessment following the primary excision biopsy confirmed the possibility of a mycetoma, based on the presence of hyphae. A formal microbiological assessment was not conducted on the original specimen and the decision was made to undertake an open biopsy from both lesions, with samples sent for culture and histopathology. Intraoperatively, multiple small black granules were apparent within the region of biopsy (Figure 3). 

**Figure 4:**
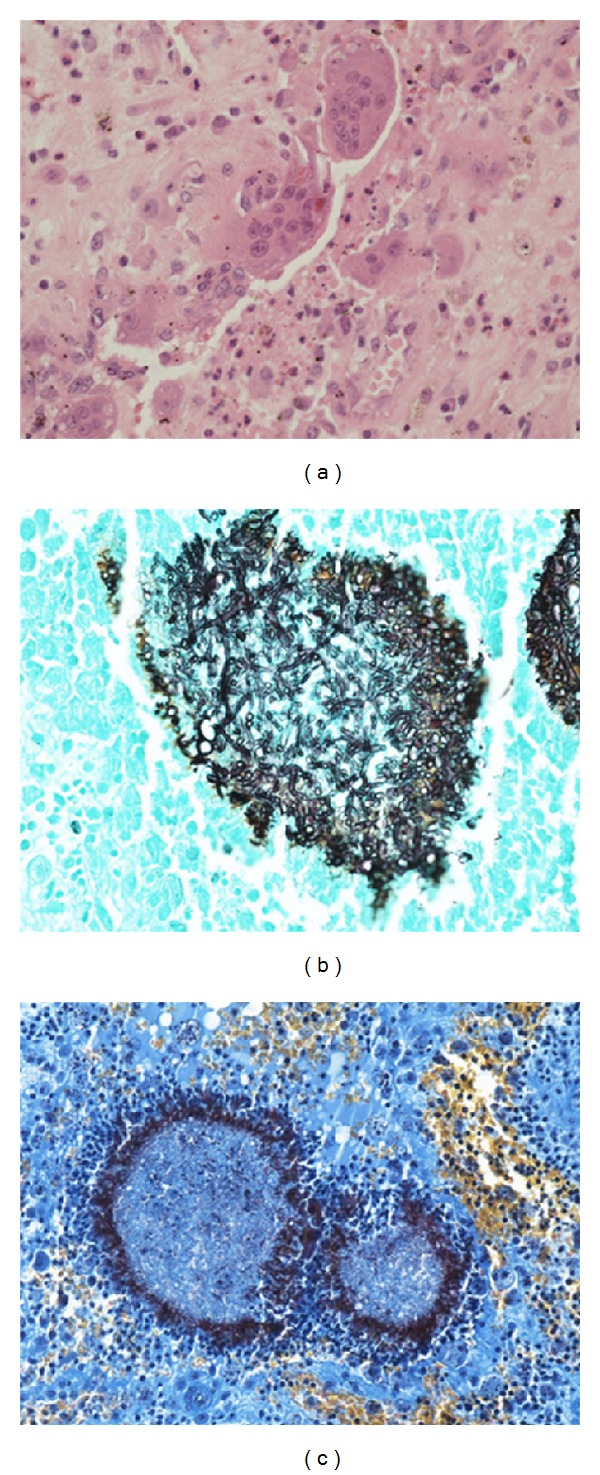
Histological findings on biopsy from the right hand. Multinucleated giant cell reaction (a). Grocott's stain demonstrating branching, septate hyphae (b). MSB stain demonstrating fungal colonies surrounded by a fibrin halo Splendore-Hoeppli's phenomenon (c).

Samples from both biopsies revealed prominent dark fungal grains in samples from the hand and significantly smaller, less dense, grains from the ankle. Histological analysis of the right hand excision biopsy revealed well-circumscribed fungal colonies (grains) comprising septate dematiaceous hyphae (confirmed by Grocott and DPAS staining), with multinucleated giant cell reaction and Splendore-Hoeppli's phenomenon (Figure 4). The diagnosis was of a mycotic abscess with acute granulomatous inflammation, consistent with eumycetoma. A sample from the right ankle demonstrated a similar morphological appearance to the hand. Culture yielded a pure growth of two morphologically and colonially distinct molds. 

Samples from the hand produced small, slow growing, gray, and domed colonies from all inoculum sites. Microscopic examination of tease mounts prepared from these colonies revealed dematiaceous hyphae but no evidence of sporulation, consistent with a presumptive identification of this organism as *Madurella grisea*. 

Microscopic examination of organisms grown from the ankle revealed abundant oval conidia formed terminally on branched long slender annelids, consistent with species of the *Pseudallescheria boydii*/*Scedosporium apiospermum* complex [4]. Sequencing of the nuclear ribosomal repeat gene cassette and of the *β*-tubulin gene [5] of this isolate confirmed that it was *S. apiospermum* sensu stricto (EMBL accession number FN600642). 

Sequences from the organism isolated from the hand lesion (EMBL accession numbers FN600643–FN600645) did not match any in the synchronized public databases but were 100% identical to sequences previously obtained at the UK National Mycology Reference Laboratory (MRL) from isolates of *M. grisea* infections acquired in the Indian subcontinent (Borman, unpublished data). No mycobacteria were observed in auramine films or on culture.

Based on antifungal susceptibility profiles compiled at the MRL for over 50 isolates encompassing 8 different causative species of dark grain mycetoma, the patient was commenced on the broad spectrum triazole voriconazole. Six months after initiation of voriconazole, there has been complete resolution of the *Scedosporium* ankle lesion but little change in the *Madurella* hand lesion.

## 3. Discussion

Gill first described the clinical presentation of eumycetoma in the natives of the Madura district of India in 1842, hence the term Madura foot. Subsequently in 1860, Carter proposed the term mycetoma and was the first to establish the fungal etiology of this disorder [6]. 

Mycetoma is characterized by the formation of grains containing aggregates of the causative organisms that may be discharged onto the skin surface through multiple draining sinuses. This process leads to the formation of microabscesses and the triad of tumefaction, suppuration, and ulceration. Left untreated, mycotic spread may occur through skin facial planes and in extreme cases may lead to bony involvement. This complication may be severely disfiguring, and involvement of the lower extremity can impede mobility.

This case presented a diagnostic challenge. There are no rapid and reliable serologic or immunologic tests useful in identifying eumycetoma due to a lack of antigens with highly specific antibody cross-reactivity. Our patient worked for 12 years sorting vegetables in a local factory, many of these vegetables originated from the Indian subcontinent, and cutaneous inoculation is the most likely explanation for his infections. Imaging was nondiagnostic but was useful in measuring disease extent and excluded bone involvement.

The initial differential diagnosis in 2008 included a ganglion cyst, vascular malformation, and neoplasia. Ultrasonographic imaging of the hand lesion demonstrated cystic regions suggestive of a ganglion, but aberrant vasculature suggested a vascular malformation. Fungal infection was not suspected, and no specimens were sent for culture from the original procedure. When he represented, the recurrence of the original right hand lesion with its apparent metastatic spread and indistinct MRI appearances leads to a clinical suspicion of a neoplastic or infective process. This unusual presentation prompted biopsy of both lesions.

A definitive diagnosis was obtained following histological analysis of surgical biopsies. Intraoperatively multiple small black granules were noted within the excised lesion. Carter subclassified mycetomatous disease into 2 categories based on granule color. He described melanoid (black, characteristically produced by *M. grisea*) or ochroid (pale-colored, characteristic of *S. apiospermum*) granules. 

This case is unique because two distinct mycotic infections have not been previously reported for an immunocompetent host. A case report by Neumeister et al. [7] discussed a patient with mycetoma due to *Exophiala jeanselmei* and *Mycobacterium chelonae*. In the reported case, however, both species were isolated from the same leg and the patient suffered from underlying idiopathic CD4+ T lymphopenia. Our case presented with no apparent immunosuppression. He was not taking any immunosuppressive medication, tested negative for HIV, and possessed normal lymphocyte subsets and immunoglobulin levels.

*S. apiospermum* in the immunocompetent host is most commonly the result of an inoculation injury. Disseminated infection may rarely follow after near drowning with presumed aspiration of a large inoculum of fungal spores. Localized or disseminated disease is well recognized in the immunocompromised host particularly opportunistic *S. apiospermum* in patients on immunosuppressive therapy after organ transplantation. Rogasi et al. [8] reported a case of *S. apiospermum* infection in a renal transplant patient who suffered recurrence of a forearm lesion with subsequent dissemination to the knee and the Achilles tendon. 

*M. grisea* has not been reported to cause disseminated infection and is seen in localised destructive subcutaneous or bone disease. Response to antifungal agents is poor in contrast to *S. apiospermum* as seen in this case where the foot lesion regressed with voriconazole. Surgery remains the mainstay of treatment for *M. grisea*. 

We have herein presented a case of eumycetoma within an immunocompetent host masquerading as simple, painless, asymptomatic swellings. This case highlights the importance of keeping a broad differential diagnosis when assessing such lumps within the surgical outpatient department. Prompt diagnosis can prevent devastating complications which may even culminate in loss of limb. Chronic fungal infection should be considered in patients from endemic regions or with occupational exposure, presenting with chronic soft tissue swelling. Biopsy and culture of ALL lesions at distinct anatomical sites should be performed and followed up cautiously.

## References

[1] Welsh O, Vera-Cabrera L, Salinas-Carmona MC (2007). Mycetoma. *Clinics in Dermatology*.

[2] Fahal AH, Suliman SH, Gadir AFA (1994). Abdominal wall mycetoma: an unusual presentation. *Transactions of the Royal Society of Tropical Medicine and Hygiene*.

[3] Mahgoub E-S (2006). Mycetoma. *Tropical Infectious Diseases*.

[4] Gilgado F, Cano J, Gené J, Sutton DA, Guarro J (2008). Molecular and phenotypic data supporting distinct species statuses for *Scedosporium apiospermum* and *Pseudallescheria boydii* and the proposed new species *Scedosporium dehoogii*. *Journal of Clinical Microbiology*.

[5] Borman A, Linton C, Miles SJ, Campbell C, Johnson E (2006). Ultra-rapid preparation of total genomic DNA from isolates of yeast and mould using Whatman FTA filter paper technology—a reusable DNA archiving system. *Medical Mycology*.

[6] McGinnis MR (1996). Mycetoma. *Dermatologic Clinics*.

[7] Neumeister B, Zollner TM, Krieger D, Sterry W, Marre R (1995). Mycetoma due to *Exophiala jeanselmei* and *Mycobacterium chebonae* in a 73-year-old man with idiopathic CD4^+^ T lymphocytopenia. *Mycoses*.

[8] Rogasi PG, Zanazzi M, Nocentini J (2007). Disseminated *Scedosporium apiospermum* infection in renal transplant recipient: long-term successful treatment with voriconazole: a case report. *Transplantation Proceedings*.

