# Erratum to “A Rare Presentation of Concurrent *Scedosporium apiospermum* and *Madurella grisea* Eumycetoma in an Immunocompetent Host”

**DOI:** 10.1155/2013/849359

**Published:** 2013-02-28

**Authors:** Vivek Gulati, Seun Bakare, Saket Tibrewal, Nizar Ismail, Junaid Sayani, Adnan Aali, Faris Kubba, William Lynn, Andrew Borman, Davinder Paul Singh Baghla

**Affiliations:** ^1^Department of Orthopaedic, Ealing Hospital NHS Trust, Uxbridge Road, Southall UB1 3HW, UK; ^2^HPA Mycology Reference Laboratory, Myrtle Road, Bristol BS2 8EL, UK; ^3^Department of Microbiology, Ealing Hospital, UK; ^4^Department of Histopathology, Ealing Hospital, UK; ^5^Ealing Hospital, UK; ^6^Mycology Reference Laboratory Center, UK

Unfortunately the submitting author omitted the pathologists involved in the case by accident. The following authors have contributed to the scientific content of the published article as follows: Adnan Aali, Faris Kubba, and William Lynn, all processed the original tissue samples, and after much research and collaboration provided us with the overall results. They produced the slides and educated the orthopaedic authors on the subject matter. Andrew Borman provided detailed analysis of the rare eumycetoma and spent copious time examining the tissue material to give us a definite diagnosis. He also oversaw the whole manuscript.

